# Penile Reconstruction With a Pedicled Suprapubic Flap After Cutaneous Necrosis Secondary to Modeling Substance Disease: A Case Report

**DOI:** 10.7759/cureus.105606

**Published:** 2026-03-21

**Authors:** Jonathan Alberto Osio-Muñoz, Carina Álvarez-Dávalos, Erick M Hernández-Mancillas, Mario Hernadez-Macillas, Sergio E Vázquez-Lara

**Affiliations:** 1 General Surgery, Centro Universitario de Ciencias de la Salud de la Universidad de Guadalajara, Guadalajara, MEX; 2 General Surgery, Universidad Autónoma de Durango, Durango, MEX; 3 General Surgery, Instituto de Seguridad y Servicios Sociales de los Trabajadores del Estado (ISSSTE), Zacetecas, MEX; 4 Plastic Surgery, Instituto de Seguridad y Servicios Sociales de los Trabajadores del Estado (ISSSTE), Guadalajara, MEX

**Keywords:** biopolymer, flap, modeling substances, penile reconstruction, ­reconstructive surgery

## Abstract

Penile cutaneous necrosis is an uncommon but severe clinical condition that may result from necrotizing infections, vascular compromise, or complications related to cosmetic procedures. The subcutaneous injection of non-medical modeling substances has been associated with chronic inflammatory reactions, fibrosis, and progressive tissue destruction that may ultimately lead to infection and skin necrosis. We report the case of a 68-year-old male with diabetes mellitus and cardiovascular comorbidities who developed extensive penile skin necrosis after subcutaneous injection of non-medical modeling substances for cosmetic augmentation. The patient presented with progressive penile pain, edema, and skin discoloration that evolved into a necrotizing soft tissue infection of the penile shaft. Radical surgical debridement was performed while preserving the corpora cavernosa and urethra. Due to the extent of the skin defect and the compromised condition of the recipient bed, immediate reconstruction was performed using a pedicled suprapubic fasciocutaneous flap based on local perforator vessels. Four weeks later, a second-stage procedure with pedicle division and definitive flap inset was successfully performed. The postoperative course was uneventful, with complete flap survival and satisfactory restoration of penile shaft coverage and contour. This case highlights the severe complications associated with non-medical penile augmentation procedures and demonstrates that pedicled suprapubic flaps represent a reliable reconstructive option for extensive penile defects, particularly in compromised surgical fields following infection or foreign-body reactions.

## Introduction

Necrotizing infections of the genital region represent uncommon but potentially life-threatening surgical emergencies characterized by rapidly progressive soft tissue destruction and systemic toxicity. Among these conditions, Fournier’s gangrene is the most recognized form of necrotizing fasciitis involving the perineal and genital fascia. Despite advances in antimicrobial therapy and critical care management, early diagnosis and prompt surgical intervention remain essential to reduce morbidity and mortality. Radical surgical debridement aimed at complete removal of devitalized tissue continues to represent the cornerstone of treatment, although it frequently results in complex soft tissue defects that require subsequent reconstructive procedures [1].

In parallel with infectious etiologies, an increasing number of complications have been reported following non-medical cosmetic penile augmentation procedures. The subcutaneous injection of foreign substances - including paraffin, silicone, mineral oils, and other non-biocompatible materials - can trigger chronic foreign-body reactions characterized by granulomatous inflammation, progressive fibrosis, and tissue destruction. This condition, commonly referred to as penile paraffinoma or modeling substance disease, may evolve over time into penile deformity, ulceration, secondary infection, and ultimately cutaneous necrosis of the penile shaft [2-4].

Once the inflammatory process or infection has been adequately controlled, reconstructive surgery becomes necessary to restore stable penile coverage while preserving both functional and aesthetic outcomes. Multiple reconstructive techniques have been described, including split-thickness skin grafts, scrotal flaps, local advancement flaps, and regional fasciocutaneous flaps. The optimal reconstructive strategy depends on several factors, including the extent of tissue loss, the vascularity and quality of the recipient bed, and patient-related factors such as systemic comorbidities and wound-healing capacity [1,5,6].

In cases involving extensive tissue loss or compromised recipient beds, vascularized regional flaps may offer clear advantages over skin grafts. Pedicled suprapubic flaps, in particular, provide well-vascularized tissue with appropriate thickness and elasticity as well as anatomical proximity to the penile shaft, making them a reliable option for resurfacing complex penile defects. Their robust blood supply allows reconstruction even in the setting of inflammation or previous infection, where graft survival may otherwise be unpredictable [1,6].

We present the case of a 68-year-old male who developed extensive penile necrosis following the injection of non-medical modeling substances for cosmetic augmentation. The patient was successfully treated with radical surgical debridement followed by staged reconstruction using a pedicled suprapubic flap, illustrating a practical reconstructive strategy for the management of complex penile defects in a compromised surgical field.

## Case presentation

**Figure 1:**
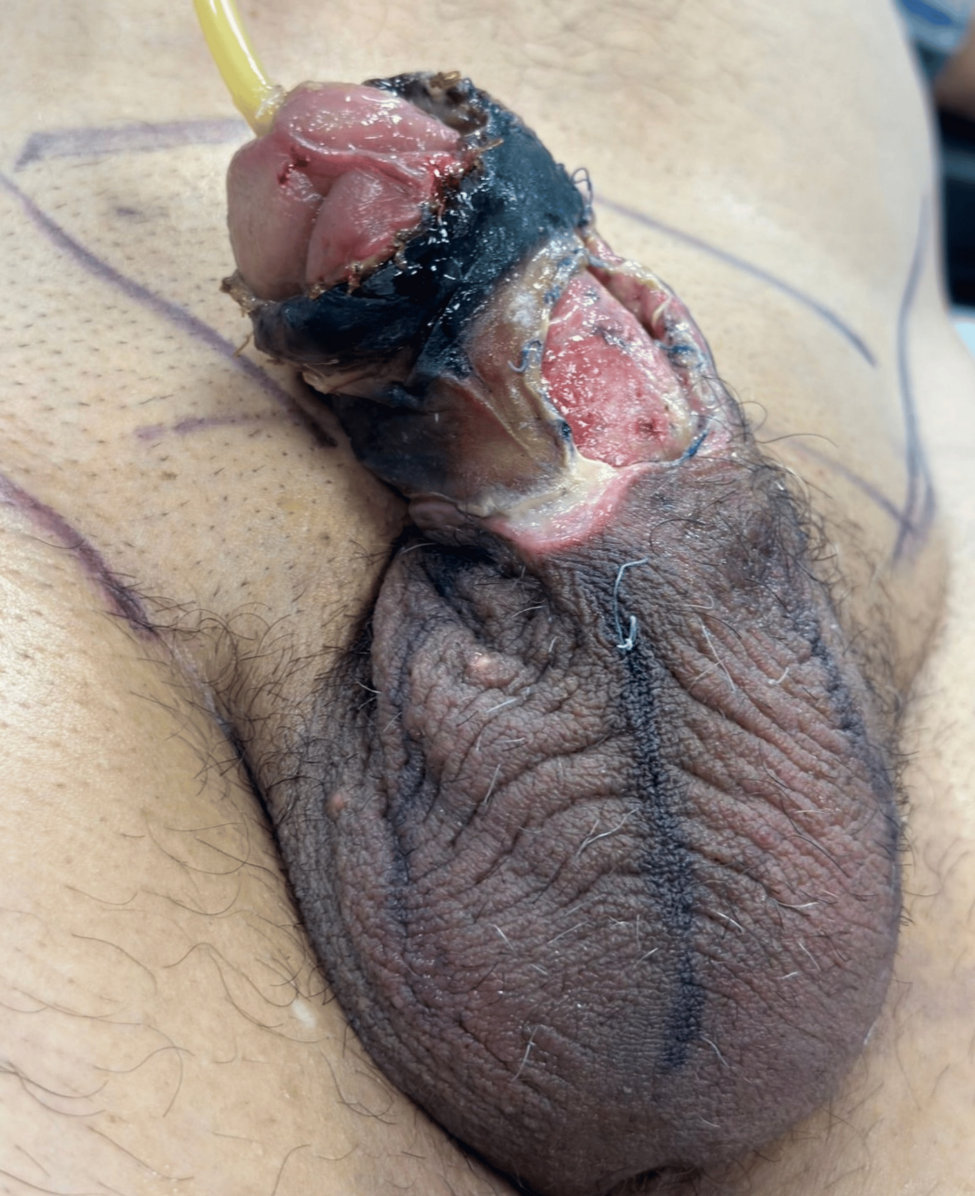
Preoperative view demonstrating extensive full-thickness penile skin necrosis predominantly affecting the ventral shaft with partial dorsal involvement. The defect shows exposure of the dartos fascia with surrounding ischemic and devitalized tissue.

A 68-year-old male, merchant by occupation, with a significant past medical history of diabetes mellitus, ischemic heart disease, and long-standing chronic venous insufficiency, presented with a severe penile soft tissue infection. The condition began on January 3, 2026, after he underwent subcutaneous penile injection of non-medical modeling substances for cosmetic augmentation, followed by a manipulation/remodeling procedure. On January 16, 2026, he developed progressive penile induration, increasing pain, edema, and cutaneous discoloration. Despite the progression of symptoms, he did not seek medical attention at that time. On January 21, 2026, he was referred to our institution and admitted with a diagnosis of penile necrotizing fasciitis (Fournier’s gangrene) secondary to modeling substance disease. Physical examination revealed extensive full-thickness necrosis of the penile skin, predominantly affecting the ventral shaft, with approximately 30% dorsal extension. The dartos fascia was exposed. There was no clinical evidence of testicular involvement, scrotal extension, palpable crepitus, or systemic hemodynamic instability at the time of admission (Figure 1).

The overall clinical presentation was consistent with modeling substance disease complicated by secondary necrotizing soft tissue infection of the penis.

Surgical technique

**Figure 2:**
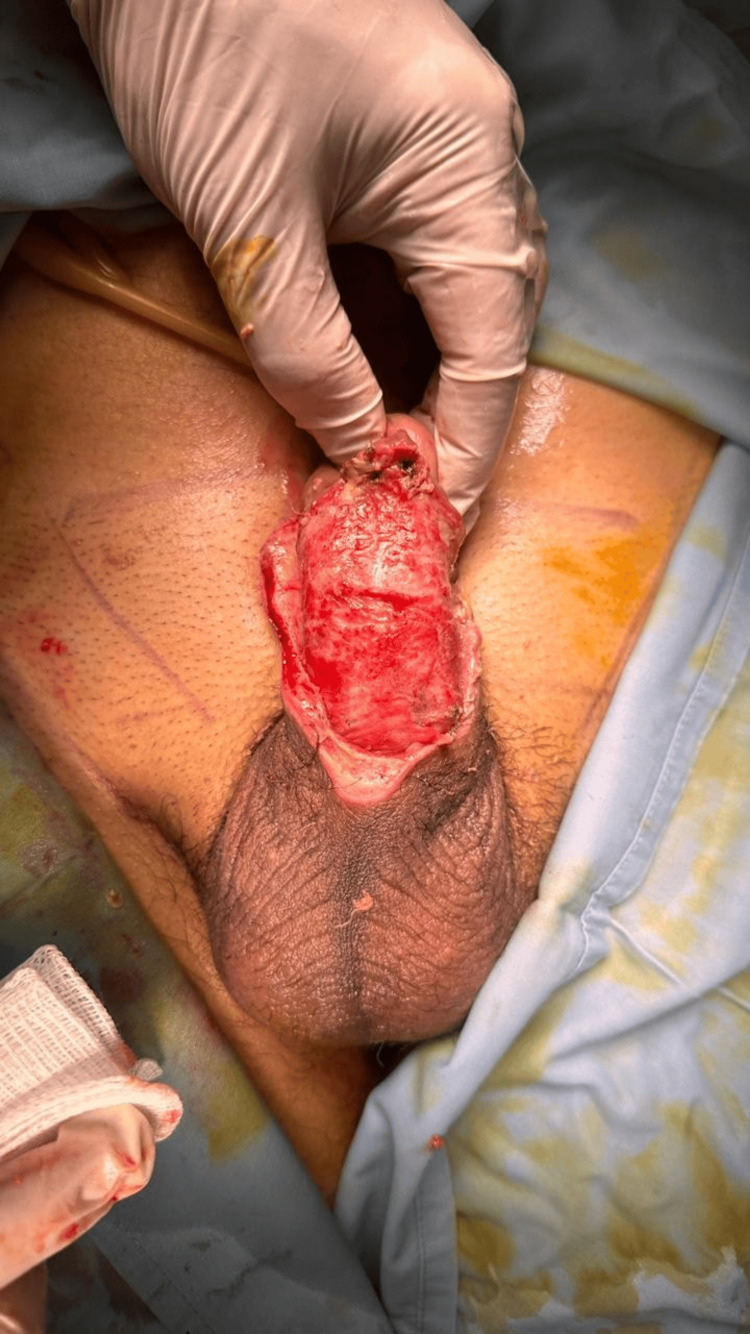
Intraoperative appearance after radical debridement for necrotizing soft tissue infection of the penis. All nonviable skin and subcutaneous tissue have been excised, resulting in near-total shaft skin loss with exposure of the underlying dartos fascia. The corporal bodies are preserved. The wound bed demonstrates adequate bleeding edges consistent with viable tissue, preparing the site for definitive reconstruction.

Under epidural anesthesia and strict aseptic protocol, a 16-Fr Foley catheter was inserted for urethral protection. Radical debridement of necrotic and devitalized tissue was performed using a No. 15 scalpel blade, excising all devitalized ventral penile skin with partial dorsal extension until healthy bleeding margins were obtained. The dartos fascia was exposed; however, the corpora cavernosa and urethra remained intact (Figure 2).

**Figure 3:**
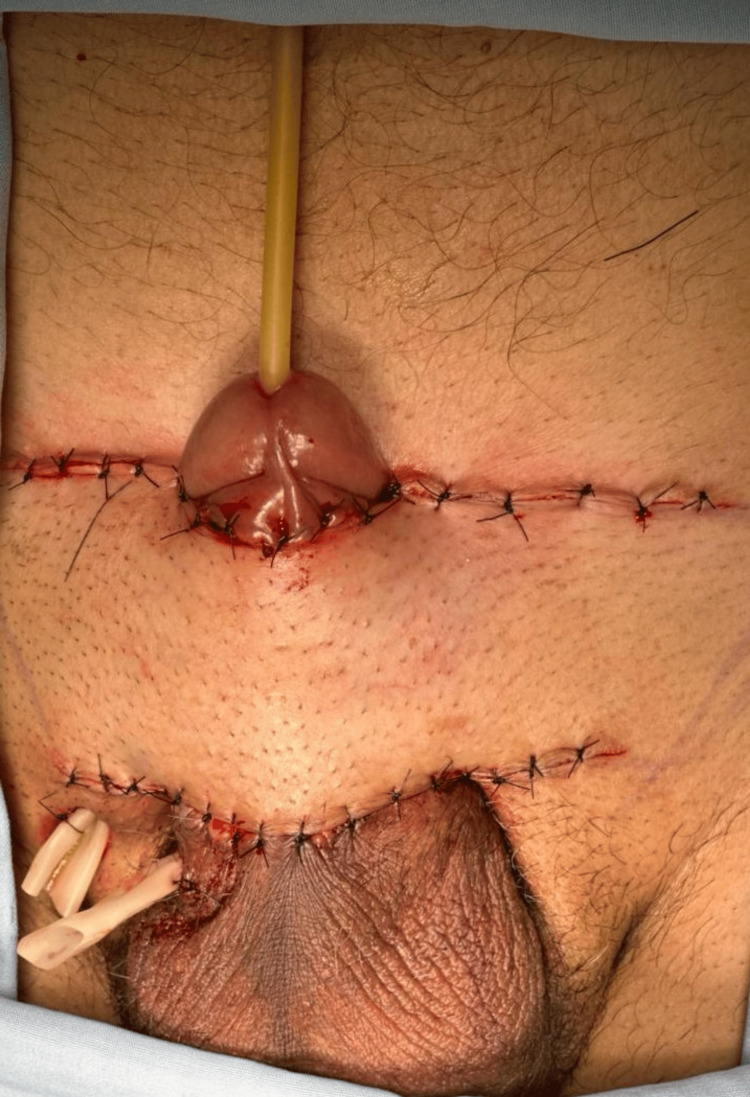
Immediate postoperative appearance after reconstruction with a pedicled suprapubic flap. The flap, designed in the suprapubic region and based on perforating vessels, was advanced inferiorly to resurface the penile shaft defect providing durable and well-vascularized coverage. The donor site was primarily closed. The flap demonstrates satisfactory color and capillary refill, consistent with adequate perfusion.

A pedicled suprapubic flap measuring approximately 12 × 3 cm was designed in the suprapubic region based on local perforators, preserving its inferior vascular pedicle. The flap was elevated and transposed inferiorly through a subcutaneous tunnel to resurface the penile shaft defect. Fixation was achieved with interrupted 3-0 nylon sutures, and layered closure of the donor site was performed (Figure 3).

**Figure 4:**
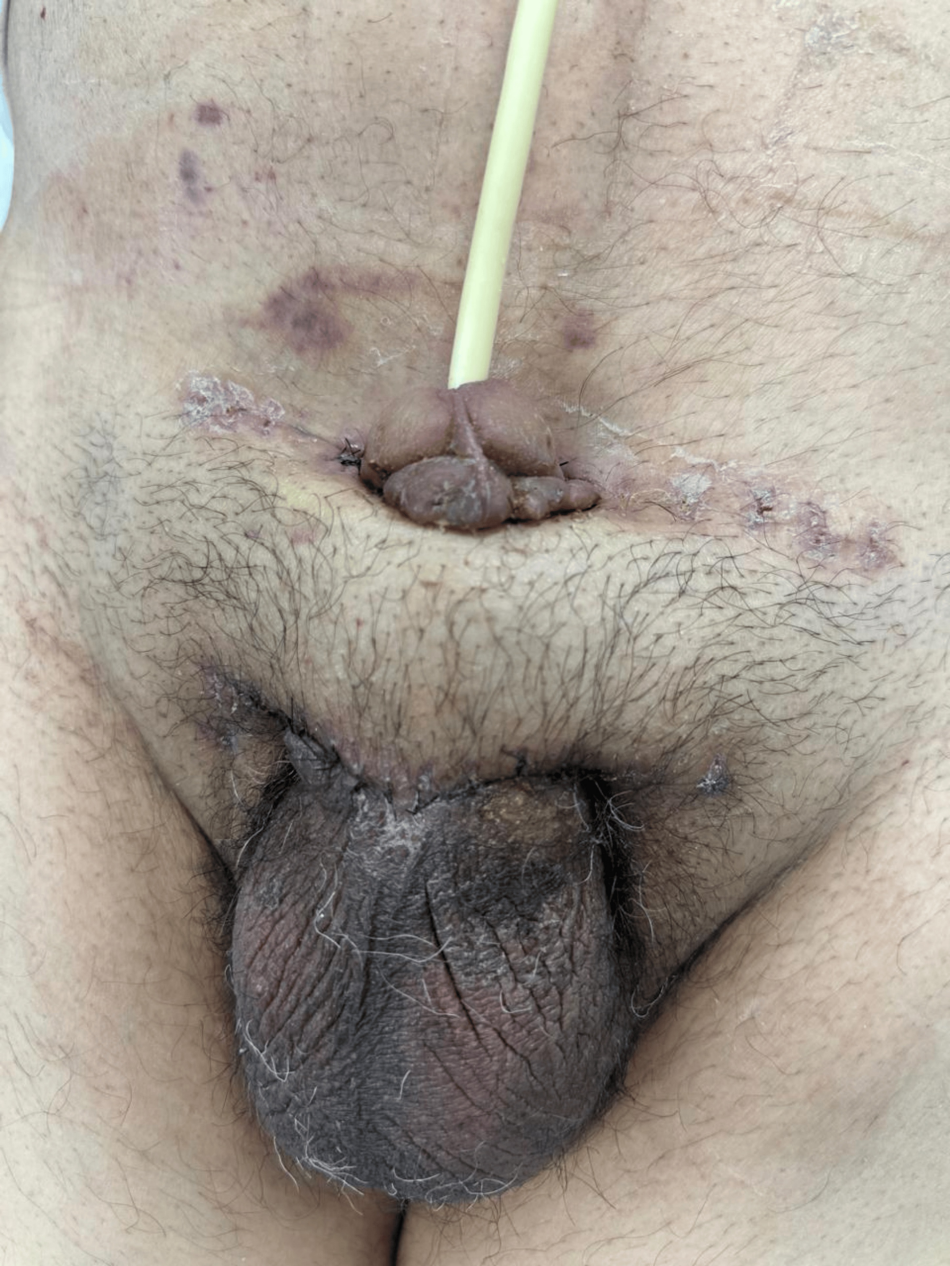
Postoperative outcome at four weeks after penile shaft reconstruction using a pedicled suprapubic flap. The flap exhibits stable vascularity, appropriate thickness, and satisfactory aesthetic contour, with no signs of ischemia or distal compromise. The donor site has healed uneventfully.

Four weeks later, a second-stage procedure was undertaken. Clinical evaluation demonstrated complete flap integration without infection, dehiscence, or ischemic compromise (Figure 4).

**Figure 5:**
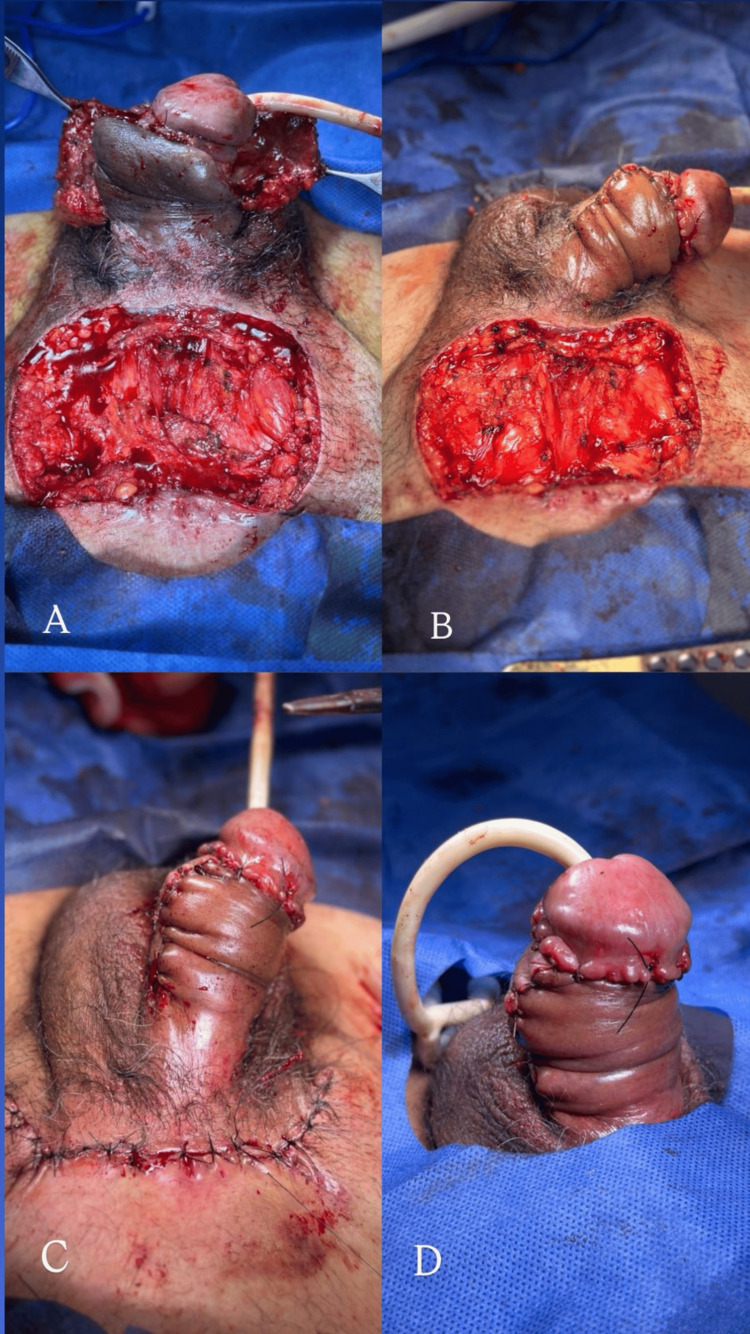
Second-stage reconstruction with pedicle division and definitive inset of the suprapubic flap. (A) Intraoperative view prior to flap division demonstrating a well-vascularized flap with complete integration to the penile shaft and a healthy recipient bed; (B) Pedicle division and release from the suprapubic donor site, with preservation of adequate distal perfusion; (C) Advancement and contouring of the flap to achieve definitive penile shaft coverage, with tension-free closure of the proximal inset; (D) Immediate postoperative appearance following completion of inset, demonstrating satisfactory flap perfusion, appropriate shaft contour, and stable soft tissue coverage.

The vascular pedicle was divided, confirming adequate distal perfusion. The flap was contoured and definitively inset to optimize shaft coverage and projection. Layered closure was performed using absorbable sutures for the subcutaneous tissue and interrupted skin sutures, restoring penile contour and length (Figure 5A-5D). A chronology of the clinical course is shown in Table 1.

**Table 1:** Chronological summary of the clinical course from subcutaneous penile modeling substance injection to staged reconstructive management.

Date	Clinical Event	Findings	Management
Day 1	Subcutaneous penile injection of non-medical modeling substances for cosmetic augmentation, followed by remodeling procedure	No immediate reported complications	No medical supervision documented
Day 13	Onset of symptoms	Progressive penile induration, increasing pain, edema, and skin discoloration	No medical treatment sought
Day 18	Hospital admission day	Extensive full-thickness necrosis of ventral penile skin with ~30% dorsal extension; exposed dartos fascia; hemodynamically stable. Clinical diagnosis of isolated penile necrotizing fasciitis (Fournier's gangrene variant) secondary to foreign modeling substance reaction.	Broad-spectrum IV antibiotics initiated at admission
Day 18	First-stage surgery	Devitalized penile skin; intact corpora cavernosa and urethra	Radical surgical debridement; 16-Fr Foley catheter placement; immediate reconstruction with pedicled suprapubic flap (12 × 3 cm)
Day 46 (Postoperative period)	Follow-up evaluation	Adequate flap perfusion; no infection, dehiscence, or ischemia	Standard wound care and clinical monitoring
Day 53 (Second-stage procedure)	Flap division and inset	Complete flap integration; adequate distal perfusion after pedicle division	Pedicle division, flap contouring, definitive inset, layered closure
Day 53 (Post-second stage)	Functional and structural restoration	Stable penile shaft coverage with satisfactory contour and projection; preserved urinary function	Outpatient follow-up

## Discussion

Penile necrosis represents an uncommon but potentially devastating clinical condition that may arise from infection, trauma, vascular compromise, or complications associated with cosmetic procedures. Among infectious etiologies, necrotizing fasciitis of the genital region, commonly known as Fournier’s gangrene, is a rapidly progressive and potentially life-threatening process characterized by extensive soft-tissue destruction and systemic toxicity. Despite advances in antimicrobial therapy and critical care management, early recognition and prompt surgical intervention remain essential to reduce morbidity and mortality. Radical surgical debridement aimed at complete removal of devitalized tissue continues to represent the cornerstone of treatment and is strongly associated with improved clinical outcomes [1].

In the present case, the underlying precipitating factor was the subcutaneous injection of non-medical modeling substances for cosmetic penile augmentation. Although these practices have been described for decades, they continue to be associated with a wide spectrum of complications. Foreign materials such as paraffin, mineral oils, and silicone can induce chronic foreign-body reactions characterized by granulomatous inflammation, progressive fibrosis, and subsequent tissue destruction. This pathological process, commonly referred to as penile paraffinoma or modeling substance disease, may ultimately result in deformity, ulceration, infection, and necrosis of the penile skin [2,3]. Previous reports have described multiple complications associated with these injections, including chronic pain, penile deformity, ulceration, erectile dysfunction, and severe soft-tissue infections. In many cases, definitive management requires surgical excision of the affected tissues because of persistent inflammatory reactions and progressive tissue damage [3,4].

Following adequate infection control through aggressive debridement and appropriate antimicrobial therapy, reconstruction of the penile shaft becomes necessary to restore both functional and aesthetic integrity. Several reconstructive strategies have been described depending on the extent of tissue loss and the characteristics of the recipient bed. These include split-thickness skin grafts, full-thickness grafts, scrotal flaps, local advancement flaps, and regional fasciocutaneous flaps. The selection of the most appropriate reconstructive technique should be individualized, taking into consideration factors such as defect size, tissue vascularity, presence of infection or fibrosis, and patient-related comorbidities that may influence wound healing [1,6].

Skin grafting remains a commonly employed technique for the management of superficial penile defects, particularly when a well-vascularized recipient bed is available. However, in cases involving chronic inflammation, fibrosis, or residual infiltration of foreign materials, graft survival may be compromised due to impaired vascularization and an unfavorable wound environment. Under these circumstances, vascularized flap reconstruction may offer several advantages over skin grafting, including improved perfusion, enhanced resistance to infection, and more reliable long-term tissue coverage.

Regional fasciocutaneous flaps have therefore emerged as valuable reconstructive options for extensive penile defects. These flaps provide well-vascularized tissue with adequate thickness and elasticity while maintaining anatomical proximity to the defect. Such characteristics allow durable coverage of large defects while preserving penile mobility and functional outcomes. Groin flaps and other regional fasciocutaneous flaps have been successfully utilized in penile reconstruction, demonstrating reliable vascularity and satisfactory functional and aesthetic outcomes in patients with complex penile soft-tissue loss [7,8].

Another important consideration in the management of complex penile defects is the potential role of staged reconstruction. A two-stage reconstructive strategy allows adequate time for infection control, optimization of the wound bed, and careful assessment of tissue viability prior to definitive flap inset. Previous reports describing staged reconstruction in patients with penile paraffinoma have demonstrated favorable cosmetic and functional outcomes, with relatively low complication rates and good long-term tissue stability [5,9].

Beyond the surgical aspects of treatment, the psychosocial consequences of penile disfigurement should not be underestimated. Loss of penile skin coverage may significantly affect body image, sexual function, and psychological well-being. Consequently, reconstructive procedures aimed at restoring normal penile anatomy may contribute not only to functional recovery but also to meaningful improvements in patient quality of life.

This case highlights the importance of individualized reconstructive planning in patients presenting with penile soft-tissue defects secondary to foreign substance injection. In selected cases, pedicled suprapubic fasciocutaneous flaps represent a practical and reliable reconstructive option, providing well-vascularized tissue coverage in situations where the recipient bed may be compromised by inflammation, fibrosis, or previous infection. Further studies evaluating reconstructive outcomes in patients with penile defects secondary to foreign substance injections are warranted to better define optimal surgical strategies and long-term functional results. Increased awareness regarding the risks associated with non-medical penile augmentation procedures is also essential to prevent severe complications such as those described in the present case.

## Conclusions

Penile necrosis secondary to foreign substance injection represents a rare but severe complication of non-medical cosmetic procedures. Early recognition and aggressive surgical debridement remain essential for infection control and prevention of further tissue loss. Following adequate debridement, reconstructive surgery is required to restore penile coverage and preserve functional outcomes. Pedicled suprapubic flaps represent a reliable reconstructive option for extensive penile defects, particularly in patients with compromised wound beds or underlying comorbidities. This case underscores the potential risks associated with non-medical penile augmentation procedures and highlights the role of vascularized regional flaps in the reconstruction of complex penile defects.
